# Effect of Cap Management Frequency on the Phenolic, Chromatic, and Sensory Composition of Cabernet Sauvignon Wines from the Central Coast of California over Two Vintages

**DOI:** 10.3390/molecules29112509

**Published:** 2024-05-26

**Authors:** L. Federico Casassa, Isabelle LoMonaco, Marcel Velasco, Dimos D. Papageorgas

**Affiliations:** 1 Wine & Viticulture Department, California Polytechnic State University, One Grand Ave., San Luis Obispo, CA 93407, USA; melasco@ymail.com (M.V.); dimosdon@protonmail.com (D.D.P.); 2 Food Science & Nutrition Department, California Polytechnic State University, One Grand Ave., San Luis Obispo, CA 93407, USA; isabellelomo@gmail.com

**Keywords:** Cabernet Sauvignon, cap management, phenolic compounds, sensory analysis

## Abstract

Cabernet Sauvignon from the California Paso Robles AVA was processed with a contrasting array of cap management frequencies, consisting of punch-down (PD) frequencies (0, 1, 2, and 3 PD/day) over two vintages, one of which the fruit was harvested at two contrasting maturity levels. Wines followed with up to 3 years of bottle aging for basic and phenolic chemistry, and the wines of the second harvest of 2020 were submitted to sensory analysis. There were almost non-existent effects due to the frequency of punch downs on parameters such as ethanol, pH, titratable acidity, lactic acid, and glucose + fructose. In 2019, the chromatic differences between different PD regimes were subtle, and minor effects of the punch-down frequency were observed for tannins and total phenolics. During the early stages of alcoholic fermentation, higher levels of all anthocyanin classes were observed in 1 PD wines and the lowest levels in 0 PD wines. The anthocyanin content of the wines of the first harvest (unripe) was 27% higher than that of the wines of the second harvest (ripe), but these differences disappeared after 3 years of bottle aging irrespective of the vintage and harvest date. Acylated anthocyanins were preferentially lost during aging, especially in 2019 wines and, to a lesser extent, in 2020 wines. In 2020, the polymeric pigment content of the wines of the second harvest was higher than in the wines of the first harvest, with 3 PD wines showing higher polymeric pigments and yellow hues than 0 and 2 PD wines after 3 years of bottle aging. Sensory analysis of the second harvest of the 2020 wines showed that the wines of all four PD regimes were perceived as drying, signifying they were perceived as equally astringent, which is consistent with comparable tannin levels on said wines. The perception of bitterness increased with the frequency of punch downs; thus, 3 PD wines showed the highest bitterness perception. It was concluded that in sufficiently warm fermentations and small volumes, phenolic extraction occurs regardless of fruit maturity and under conditions of minimum mixing.

## 1. Introduction

The post-modern understanding of the maceration process during red winemaking entails adjusting variables such as temperature and cap management regime to a specific variety, location, climate, and/or style of wine sought. Maceration is a key stage in red winemaking, and it is defined as the time during which the fermentation solids (i.e., skins, seeds, and stems, when present) are in contact with the fermenting must. This continues until the process is deemed completed at the time of pressing. The release of CO_2_ from the alcoholic fermentation creates pressure, causing the upward movement and accumulation of fermentation solids atop of the fermenter, with this pressure being counter to the atmospheric pressure. This counterpressure system creates what is known as the cap, which is composed of compressed skins and seeds and thus a gradient following Fick’s second law of diffusion (i.e., the rate of diffusion is a function of the solute concentration gradient and the internal diffusion coefficient) is created underneath the aforementioned cap [1]. Diffusing materials into the fermenting must include key components for wine sensory composition, including anthocyanins, tannins, aromas, and aroma precursors [2], some of which further react between each other upon release into the fermenting must. A specific set of these reaction products include polymeric pigments, which result from reactions between anthocyanins and tannins [3]. Anthocyanins are pigments responsible for wine color and typically reach peak extraction after 5 to 6 days post-crushing, which is followed by a drop from peak concentration [4]. The latter is due to a combination of different phenomena including degradation, adsorption [5], and the formation of anthocyanin-derived pigments [3]. Tannins are the major contributors to the tactile sensation of astringency and diffuse into the fermenting must from seeds and skins, but each tissue shows specific rates of extractions. For example, in Cabernet Sauvignon fermentations, increasing the must temperature affects the rate of extraction of skin-derived phenolics but not the final concentration, whereas increasing the temperature increases both the rate and final concentration for seed-derived phenolics [6]. Mathematical modeling has shown that seed phenolics are extracted following zero-order kinetics, and this likely results from multiple competing phenomena on seed-derived phenolic extraction [7].

The diffusion gradient that is established between the cap and the fermenting must has been empirically considered by winemakers as a key feature to manage during maceration. In effect, if dissolution and diffusion of sensory-relevant materials are to be maintained, the interphase between the fermenting must and the cap should be disrupted. The latter constitute the underpinning reason behind the implementation of a cap management protocol. Cap management techniques vary widely depending upon variety, region, winery logistics and equipment, fermenter volume, geometry, and materials, among other factors. Under standard red winemaking conditions, the cap is typically disrupted by mechanical actions such as punch downs, pump overs [8], and rack-and-return actions [9], though other techniques rely on minimizing this diffusion gradient by keeping the cap submerged in the fermenting must [10].

The mechanical act of punch downs, and its frequency, arguably represents the simplest way to disrupt the cap. This frequency can typically range from zero (i.e., no cap management) to up to two punch downs/day. A work in Merlot, whereby the fruit was processed with two punch downs/day (dubbed traditional maceration), half-plunge (i.e., starting the punch-down regime after Day 7 post-crush), and no plunge (i.e., no punch down), showed that the concentration of total phenolics, pigmented tannins, and free anthocyanins were higher in no plunge wines, followed by half-plunge, with the wines made by traditional maceration showing comparatively lower concentrations [11]. The authors ascribed these results to “increased exposure to oxygen during mechanical cap management but also increased disruption of berry solids, leading to more adsorption and oxidation of the extracted phenolics”. However, because the latter research was carried out in 12 kg fermenters, the results may not extrapolate to fermenters of larger volume, where the dynamics of heat buildup and heat release may vary vastly relative to those observed in small-volume ferments. Recent reports on the effect of no cap management both at research and industrial scale have focused on both phenolic and volatile chemistry. Pinot noir wines from two different clones (UDC 23 and 828) were produced at industrial scale (1.5 tons fermenters) with two punch downs throughout the alcoholic fermentation (control), whereas minimal intervention (MI) wines did not receive punch downs until the completion of alcoholic fermentation [12]. The results indicated minor differences in the general phenolic composition between treatments, with anthocyanins being slightly higher in control wines and tannins being higher in MI wines. The authors ascribed tannin extraction in the absence of cap management to the mixing effect of CO_2_ and the increased pressure and temperature build-up in the undisturbed cap [12]. 

The literature cited above suggest that, under specific conditions (e.g., volume and geometry of the fermentor), phenolic extraction may occur despite the absence of physical mixing and disruption of the cap. However, it is not clear how the frequency of disruption and mixing affects phenolic extraction and chromatic development. Furthermore, fruit ripeness can affect both the extractability and concentration of major phenolic classes. This is because during and over ripening, the progressive de-pectination of cell walls may allow for an easier deconstruction of grape pomace during maceration, thereby enhancing the phenolic extraction in wine [13]. Per the latter, it is also of interest to explore the combined effect of selected frequencies of punch downs in fruit of contrasting ripeness. 

In the present work, Cabernet Sauvignon fruit was processed with a contrasting array of punch-down frequencies, ranging from zero to four punch downs/day over two consecutive vintages. Importantly, in one of the vintages, the fruit was harvested with two contrasting maturity levels to explore the potential interactions between fruit ripeness and punch-down frequency. An initial working hypothesis was that the frequency of punch downs would be a lesser factor on phenolic extraction when applied to riper fruit than when applied to less ripe fruit. Herein, the detailed phenolic composition, chromatic characteristics, and sensory composition of the resulting wines is reported.

## 2. Results and Discussion

### 2.1. Basic Fruit and Wine Chemistry 

Table 1 shows the basic chemical composition of the grapes at harvest time. In 2019, the fruit was harvested at a single time point, targeting Brix levels normally aimed at in Premium Cabernet Sauvignon wine production. In 2020, two harvest dates were established, one to signify an early harvest and the other to signify a commercial standard harvest. Of note was that both vintages were vastly different, whereby 2019 was cooler than average and 2020 was warmer than average (Supplementary Materials Figure S1). Consequently, the second harvest in 2020 occurred earlier, albeit reaching similar Brix levels than in 2019. The colder nature of the 2019 vintage was also reflected in the lower pH and higher titratable acidity when comparing the 2019 fruit to the 2020 fruit, even despite comparable sugar levels at harvest (Table 1).

**Figure 1:**
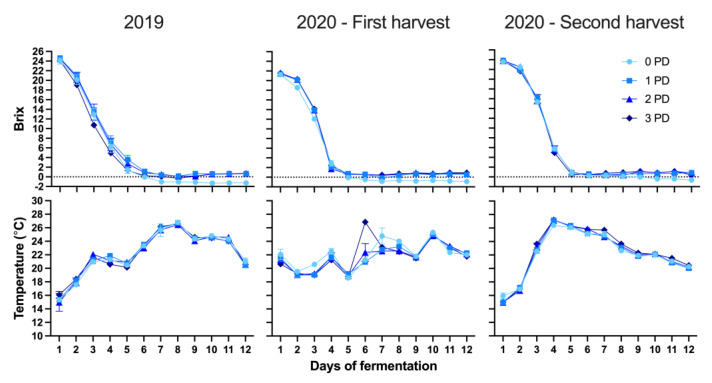
Evolution of the sugar consumption measured as Brix (**top panel**) and temperature (**bottom panel**) during the alcoholic fermentation in Cabernet Sauvignon wines fermented with different cap management (punch-down) frequencies over two consecutive vintages. Each data point represents the average of three tank replicates (n = 3).

Table 2 shows the basic chemical composition of the wines of both vintages and when under different harvest times. Alcohol levels by volume (ABV) ranged from 14% ABV on average in 2019 to 11% ABV on average in the first harvest of 2020, and to 12.5% ABV on average on second harvest of 2020. This variation afforded the possibility to gauge the effect of the punch-down regime under a wide range of alcohol levels. There were almost non-existent effects of the frequency of punch downs on parameters such as ethanol, pH, titratable acidity, lactic acid, and glucose + fructose (Table 2). However, in the present study, all the wines were submitted to very similar temperature regimes irrespective of the frequency of punch downs (Figure 1). This likely explains the lack of effect of the punch-down regime on the basic chemistry of the wines that was herein observed. It also showed that the frequency of punch downs did not generally affect the basic chemistry of the wines, an outcome that has been also observed in Pinot noir wines produced with limited (minimal intervention) cap management [12]. Additionally, the lack of effect of punch-down frequency on parameters such as acetic acid and glucose + fructose could be because, in addition to very similar temperature profiles, the wines showed very comparable rates of sugar consumption during alcoholic fermentation (Figure 1). It should be noted, however, that different outcomes may be observed when using a yeast strain different than the one that was herein employed (D 20, Enartis). 

### 2.2. Phenolic and Chromatic Composition of the Wines

#### 2.2.1. 2019 Vintage 

**Figure 2:**
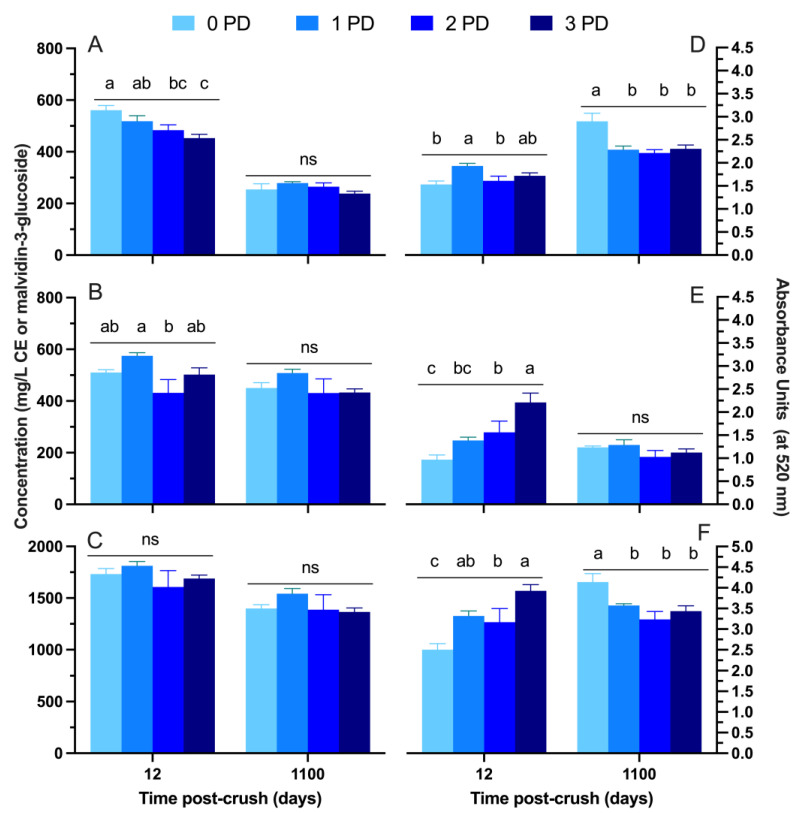
Concentrations at Day 12 post-crushing (pressing) and at Day 1100 post-crush (3 years after crushing) of (**A**) anthocyanins; (**B**) tannins; (**C**) total phenolics; (**D**) small polymeric pigments; (**E**) large polymeric pigments; and (**F**) total polymeric pigments in Cabernet Sauvignon wines fermented with different cap management (punch-down) frequencies. The wines were of 2019 vintage. Different letters within the same time point indicate significant differences between the treatments as established by Fisher’s LSD, with a significance level of *p* < 0.05. ns: not significant. Error bars indicate the standard error of the mean (n = 3).

**Figure 3:**
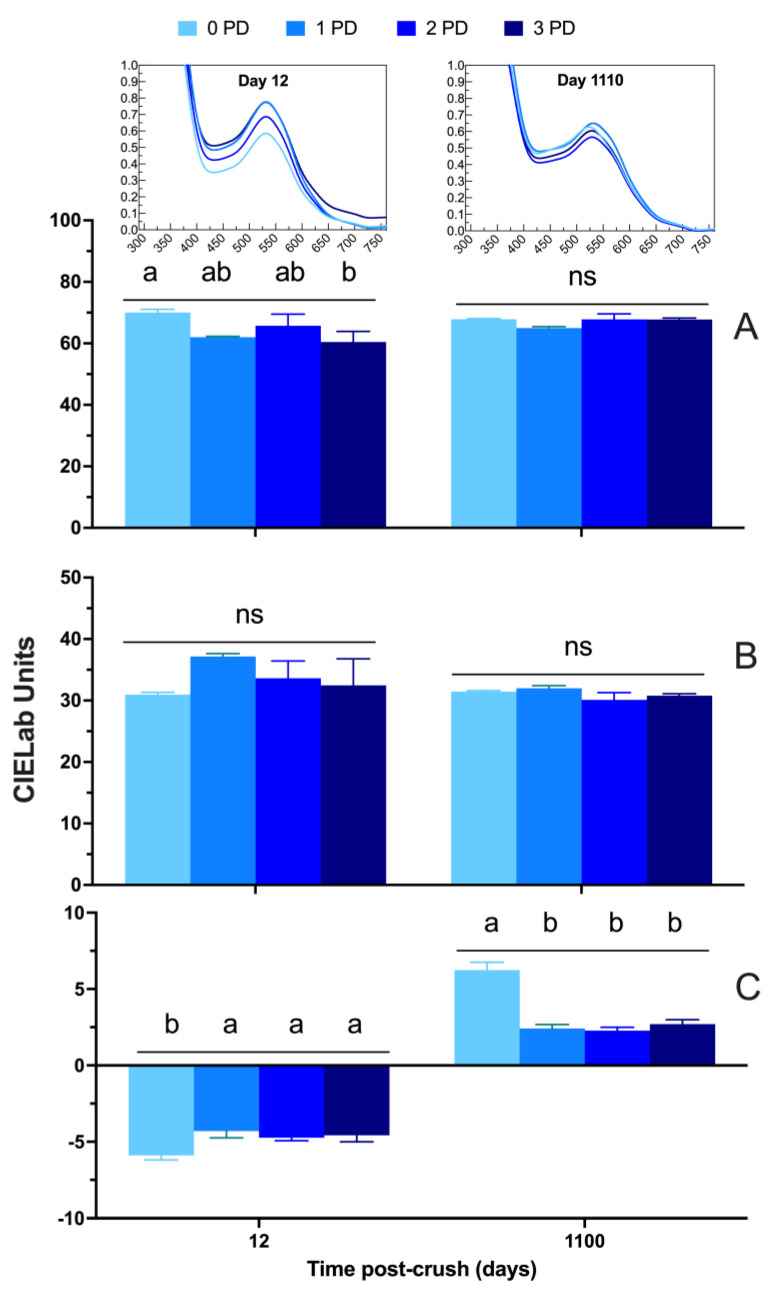
Chromatic composition of the wines at Day 12 post-crushing (pressing) and at Day 1100 post-crush (3 years after crushing) of (**A**) lightness; (**B**) a* (red color when positive); and (**C**) b* (yellow color when positive) in Cabernet Sauvignon wines fermented with different cap management (punch-down) frequencies. Top panel shows the full visible spectrum scans (between 300 to 750 nm) of the wines at Day 12 and 1100 post-crush. The wines were of 2019 vintage. Different letters within the same time point indicate significant differences between the treatments as established by Fisher’s LSD, with a significance level of *p* < 0.05. ns: not significant. Error bars indicate the standard error of the mean (n = 3).

Figure 2 and Figure 3 show the phenolic and chromatic composition, respectively, of the wines of the 2019 vintage at pressing and after 3 years post-crushing. At pressing (Day 12), the anthocyanins were higher in the 0 PD wines than in the 3 PD wines. This indicates that the complete absence of cap management leads to a higher initial extraction of anthocyanins than a high frequency of punch downs. However, this was not reflected in the chromatic composition of the wines (Figure 2), whereby full-spectrum scans showed less color in the 0 PD wines (Figure 3, insets) and slightly higher lightness (i.e., less saturation) than the 3 PD wines at pressing (Figure 3A). It is likely that, under the conditions of 0 PD wines, a portion of the anthocyanin pool was in the hemiacetalic or colorless form, as observed under reductive conditions [13], thus explaining the actual less perceived color in the full-spectrum scans. However, these colorless adducts may turn into the colored flavylium cation under the conditions of the anthocyanin analysis determination, thus explaining the higher anthocyanins (but lower color) in the 0 PD wines. The reductive conditions in the 0 PD wines of the present study, however, can only be inferred as the oxidation–reduction potential was not monitored. 

It is also worth noting that these chromatic differences were subtle at best, and this was further compounded by the fact that the minor effects of the punch-down frequency were also observed for tannins (Figure 2B) and total phenolics (Figure 2C). Notably, the rather unremarkable effect of the frequency of the punch downs on the basic phenolic composition of the wines at pressing sits at odds with empirical winemaking observations. That is, a higher frequency of physical mixing should lead to higher phenolic extraction. Recent observations suggest that a higher frequency of mixing may lead to phenolic losses due to chemical and or enzymatic oxidations [12]. Such losses may be minimized under conditions of no mixing—akin to the 0 PD treatment. Phenolic extraction in the absence of cap management may be explained as follows: in fermentors of relatively small volume, and under conditions of sufficiently high fermentation temperature, convection movements may facilitate physical mixing thereby allowing for sufficient phenolic extraction. Furthermore, an undisturbed cap may build more pressure than one that is frequently disrupted, as the upward pressure of CO_2_ release compresses the cap against atmospheric pressure. However, the potential detrimental effect of oxygen dissolution derived from a high frequency of punch downs on phenolic degradation still needs to be unequivocally ascertained. The measurement of the evolution of the oxidation–reduction potential of fermentations under such contrasting conditions should shed some light into this observation. After 3 years post-crushing, the frequency of punch downs resulted in no differences in the levels of anthocyanins, tannins, total phenolics (Figure 2A, Figure 2B, and Figure 2C, respectively), lightness, and a* (Figure 3A and B, respectively). Small polymeric pigments, which include small molecular weight pigments such as cycloaddition and acetaldehyde cross-linked pigmented products [14], and total polymeric pigments were higher in the 0 PD wines (Figure 3D and 3F, respectively) after 3 years post-crushing. This result aligns with the fact that the 0 PD wines showed higher values of b* (yellow component) after 3 years post-crushing. Indeed, it was assumed that polymeric pigments, which were higher in the 0 PD wines at Day 1100 post-crush, display yellow and red-brick hues as opposed to the purple hues observed in intact anthocyanins [15]. Therefore, the long-term positive effect of the no cap management treatment, especially regarding the polymeric pigment composition, was herein put into evidence.

**Figure 4:**
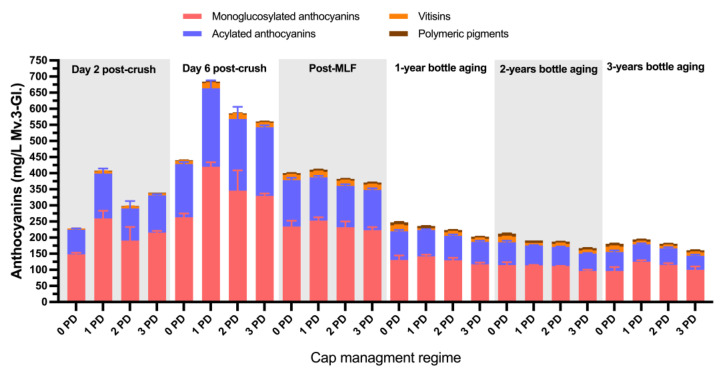
Evolution during the winemaking and bottle aging of monoglucosylated and acylated anthocyanin derivatives, vitisins, and polymeric pigments in Cabernet Sauvignon wines fermented with different cap management (punch-down) frequencies. The wines were of 2019 vintage. Error bars indicate the standard error of the mean (n = 3).

Figure 4 shows the detailed anthocyanin composition of the 2019 wines throughout the winemaking and up to 3 years of bottle aging. For simplicity, the anthocyanins were grouped in classes such as monoglucosylated, acylated, vitisins, and polymeric pigments. The anthocyanins peaked at Day 6 post-crush, with notoriously higher levels being recorded for all anthocyanin classes in the 1 PD wines and the lowest levels recorded in the 0 PD wines. However, after malolactic fermentations, the difference between treatments subsided almost completely. These results suggest that the anthocyanins were extracted at a lower rate in the 0 PD wines, but also their losses occurred at a lower rate relative to the treatments that received cap management. The relatively higher early anthocyanin extraction in the 1 PD wines followed by the steepest decline could be, at least, partially accounted for by the incorporation of these anthocyanins into polymeric pigments. Indeed, at Day 12 post-crushing, the treatments that received cap management did relatively show higher levels of SPP and LPP to the 0 PD wines (Figure 2). After 3 years of bottle aging, the levels of most anthocyanin classes were virtually the same, and this was also reflected in the color of the wines at this stage (Figure 3). 

At Day 6 post-crushing, monoglucosylated anthocyanins accounted for 60% of the total anthocyanin composition, whereas acylated anthocyanins accounted for 37% of the total anthocyanin composition. After 3 years of bottle aging, monoglucosylated anthocyanins accounted for 61% of the total anthocyanin composition, but, contrastingly, acylated anthocyanins accounted for 29% of the total anthocyanin composition. This suggests that acylated anthocyanins were preferentially lost during aging, with the understanding that a portion of them may have become incorporated into the polymeric pigments (Figure 2). The preferential loss of acylated over monoglucosylated anthocyanins during aging has been previously observed [4], which is also in agreement with the results herein presented.

#### 2.2.2. 2020 Vintage 

**Figure 5:**
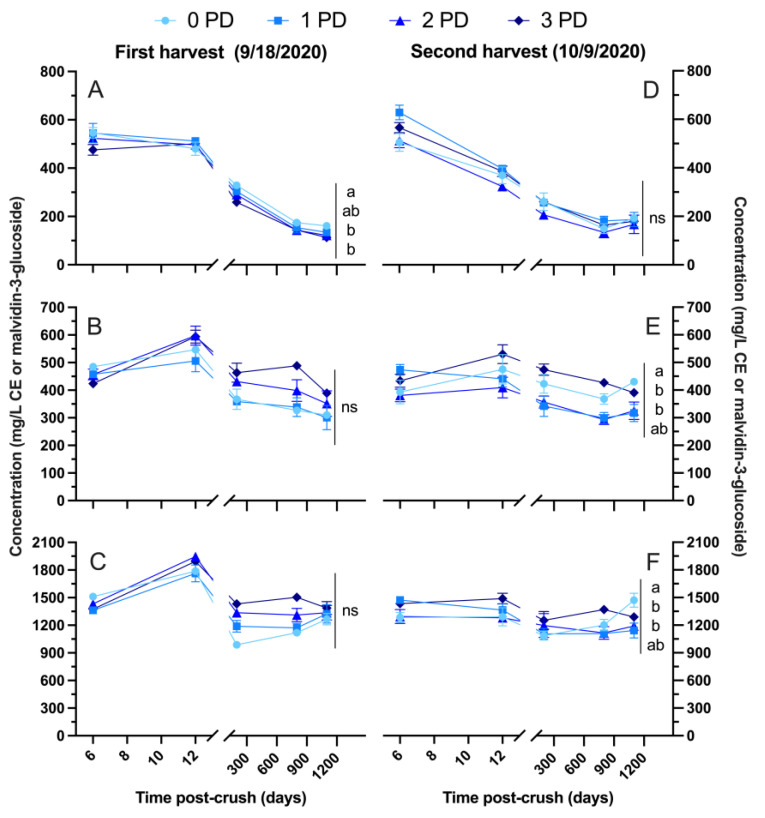
Evolution during the winemaking and up to 1100 days post-crushing (3 years) of (**A**,**D**) anthocyanins; (**B**,**E**) tannins; and (**C**,**F**) total phenolics in the Cabernet Sauvignon wines harvested at two maturity levels (first and second harvest) and fermented with different cap management (punch-down) frequencies. The wines were of 2020 vintage. Different letters at Day 1100 post-crush indicate significant differences between the treatments as established by Fisher’s LSD, with a significance level of *p* < 0.05. ns: not significant. Error bars indicate the standard error of the mean (n = 3).

**Figure 6:**
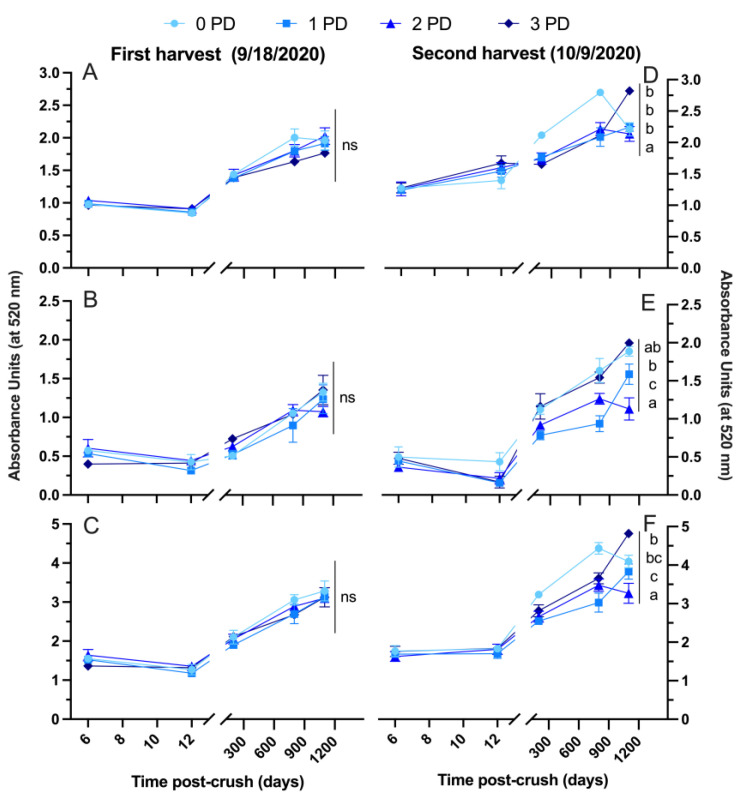
Evolution of the polymeric pigment content of the wines during winemaking and up to 1100 days post-crushing (3 years) of (**A**,**D**) small polymeric pigments; (**B**,**E**) large polymeric pigments; and (**C**,**F**) total polymeric pigments, in Cabernet Sauvignon wines harvested at two maturity levels (first and second harvest) and fermented with different cap management (punch-down) frequencies. The wines were of 2020 vintage. Different letters at Day 1100 post-crush indicate significant differences between the treatments as established by Fisher’s LSD, with a significance level of *p* < 0.05. ns: not significant. Error bars indicate the standard error of the mean (n = 3).

Figure 5 and Figure 6 show the evolution of the basic phenolic and polymeric pigment composition, respectively, of the wines of both harvests in 2020. As with the 2019 vintage, the wines followed up to 3 years (1100 days) post-crushing. Anthocyanins, tannins, and total phenolics peaked at the pressing of the wines in the first harvest (Figure 5A, Figure 5B, and Figure 5C, respectively), whereas these phenolics were generally higher at Day 6 post-crushing in the second harvest. In the first harvest, anthocyanins dropped by about 65% from pressing to 1100 days post-crushing, with the 0 PD wines retaining more anthocyanins than the 2 PD and 3 PD wines (Figure 5A). A very similar ~65% drop from pressing to Day 1100 post-crushing occurred in the wines of the second harvest, but no differences were evident at the last sampling point. Tannins (Figure 5B) and total phenolics (Figure 5C) evolved similarly in the wines of the first harvest, with no overall differences after 3 years post-crushing. Tannins and total phenolics were higher in the 0 PD wines of the second harvest, especially in comparison with the 1 PD and 2 PD wines.

As expected, the small (SPP), large (LPP) and total polymeric pigment content of the wines increased gradually during bottle aging, whereas there were no treatment effects on the SPP, LPP and total polymeric pigments observed in the wines of the first harvest (Figure 6A, Figure 6B, and Figure 6C, respectively) as the general polymeric pigment content of the wines of the second harvest was higher than in the wines of the first harvest. Also notable, the 3 PD wines showed a higher total polymeric pigment content than the 0 PD and 2 PD wines after 1100 days post-crushing (Figure 6F). These results directly counter the initial working hypothesis. The hypothesis was that punch-down frequency will play a more critical role on the phenolic extraction in unripe fruit. This was thought because unripe fruit is generally believed to have lower phenolic extractability, especially in terms of skin-derived phenolics [16]. Thus, a higher frequency of punch downs should, at least, partially counter this allegedly lower extractability in unripe fruit. Counter to this, a slightly higher tannin extraction in the 0 PD wines, and higher polymeric pigment content in the 3 PD wines of the second harvest was observed. It is thus possible that the differences in phenolic extractability within the two ripeness levels may have been masked by a sufficiently high fermentation temperature (Figure 1). This higher fermentation temperature, which was largely unaffected by the punch-down regime, was also able to counter any effect of the frequency of punch downs on phenolic extraction. The present results suggest that in sufficiently warm fermentations and small volumes, phenolic extraction occurs regardless of fruit maturity (at least within the ripeness levels assessed in the present study), even in the absence of cap management. 

**Figure 7:**
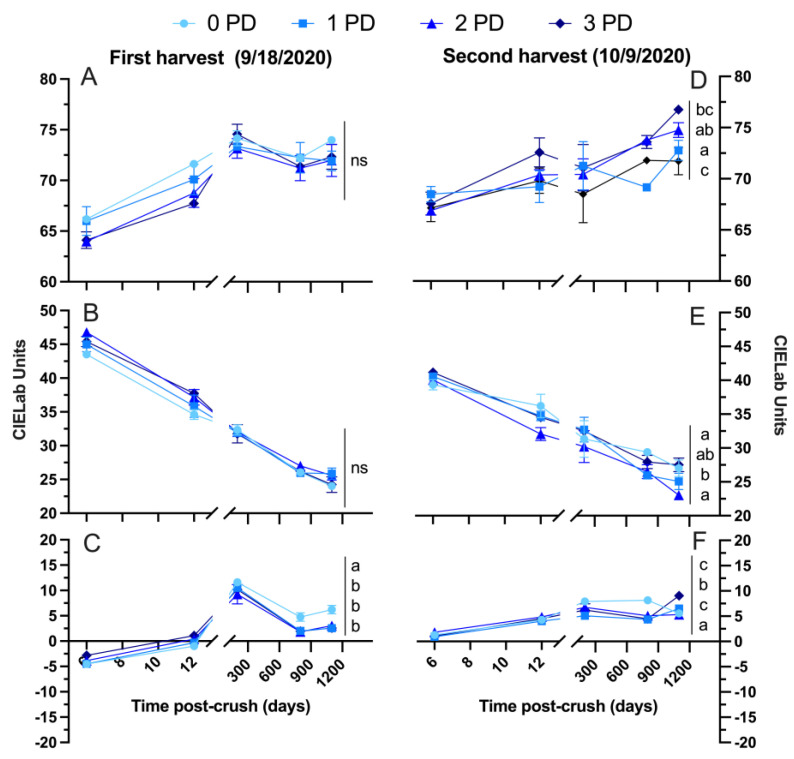
Evolution of the chromatic composition of the wines during winemaking and up to 1100 days post-crushing (3 years) of (**A**,**D**) lightness; (**B**,**E**) a* (red color when positive); and (**C**,**F**) b* (yellow color when positive) in the Cabernet Sauvignon wines harvested at two maturity levels (first and second harvest) and fermented with different cap management (punch-down) frequencies. The wines were of 2020 vintage. Different letters at Day 1100 post-crush indicate significant differences between the treatments as established by Fisher’s LSD, with a significance level of *p* < 0.05. ns: not significant. Error bars indicate the standard error of the mean (n = 3).

Figure 7 shows the detailed chromatic composition of the 2020 wines throughout winemaking and up to 1100 days post-crushing. Lightness increased steadily in all the wines from Day 6 and onward, signifying the general loss in saturation, which was especially observed during and after malolactic fermentation. The wines of the first harvest generally showed no effect of the cap management regime on their respective chromatic composition, which is in line with the phenolic data (Figure 6). At Day 1100 post-crushing, there were no differences between the punch-down treatments on lightness (Figure 7A) and the red color component, a* (Figure 7B). However, b*, the yellow component, was higher in the 0 PD wines, signifying potentially more prominent signs of color evolution on the said wines relative to the 1, 2, and 3 PD wines (Figure 7C). 

There were chromatic differences for all the three parameters, L*, a*, and b*, in the wines of the second harvest. However, these differences were relatively minor. Lightness (Figure 7D) and b* (Figure 7F) were the highest in the 3 PD wines. This means these wines were relatively lower in color saturation and showed more yellow hues than the wines of the remaining treatments. This finding aligns with the observation that the 3 PD wines showed a higher total polymeric pigment content than the 0 PD and 2 PD wines after 1100 days post-crushing (Figure 6F). The red color component, a*, was higher in the 0 PD and 3 PD wines, whereas the 2 PD wines showed the lowest a* values (Figure 7E). These differences between, for instance, the 0 PD wines and 2 PD wines were of 4 CIELab units in favor of the 0 PD wines; thus, it is likely to be of sensory relevance.

**Figure 8:**
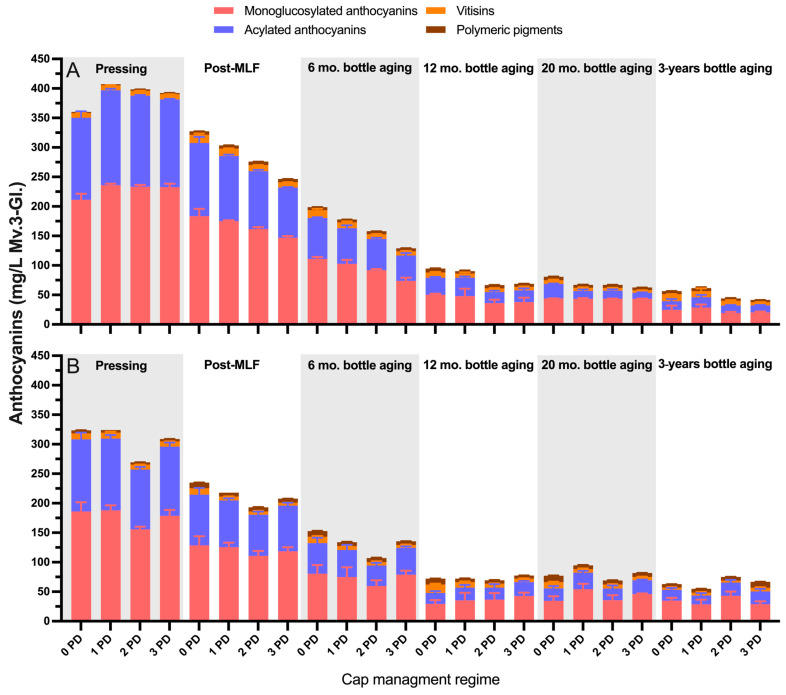
Evolution during the winemaking and bottle aging of monoglucosylated and acylated anthocyanin derivatives, vitisins, and polymeric pigments in the Cabernet Sauvignon wines harvested at two maturity levels and fermented with different cap management (punch-down) frequencies. (**A**) first harvest, top panel; (**B**) second harvest, bottom panel. The wines were of 2020 vintage. Error bars indicate the standard error of the mean (n = 3).

Figure 8A,B show the detailed anthocyanin composition of the wines of the first and second harvest of 2020, respectively, throughout the winemaking and up to 3 years of bottle aging. Interestingly, at pressing, the general anthocyanin content of the wines of the first harvest (unripe) was on average 27% higher than that of the wines of the second harvest (ripe). This reduction in anthocyanins in the wines of the second harvest, which occurred 21 days later relative to the first harvest, may reflect the effect of a series of heat waves, as well as covered skies due to the smoke fires in Monterey County in 2020, on anthocyanin composition. While the smoke did not affect the grapes, it could have impaired anthocyanin accumulation, which likely explains the present results. 

At pressing, the monoglucosylated and acylated anthocyanins accounted for 59% and 39%, respectively, of the total anthocyanin content of the wines of the first harvest, and for 58% and 39%, respectively, of the wines of the second harvest. Starting post-malolactic fermentation, there was a trend of slightly higher levels of most anthocyanin classes in the 0 PD wines of both harvests, but these differences subsided after 3 years of bottle aging (Figure 8A,B). Relative to the 2019 anthocyanin data, the wines of the first harvest showed a lower proportion of monoglucosylated and acylated anthocyanins (46% and 28%, respectively) and a higher relative predominance of polymeric pigments and vitisins (Figure 8A) after 3 years of bottle aging. A similar trend was observed in the wines of the second harvest (52% and 30% for the monoglucosylated and acylated anthocyanins, respectively). 

#### 2.2.3. Sensory Analysis

**Figure 9:**
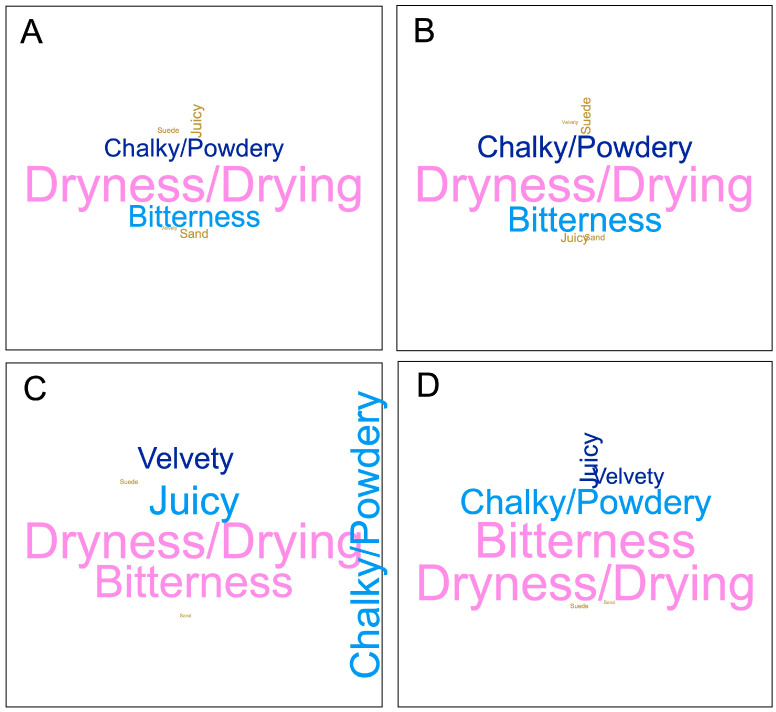
Word clouds depicting, by relative size, the frequency of citation of Cabernet Sauvignon wines fermented with different cap management (punch-down) frequencies, as assessed by a trained sensory panel (n = 13): (**A**) 0 PD; (**B**) 1 PD; (**C**) 2 PD; and (**D**) 3 PD. Second harvest, 2020 vintage.

Figure 9 shows an overview of the sensory composition of the wines of the second harvest from the 2020 vintage. These wines were selected over those at the first (i.e., unripe) harvest as they were more representative of the harvest decisions in the commercial production of Premium Paso Robles AVA Cabernet Sauvignon in California. Word clouds allow one to visualize the frequency of citation of a given descriptor, and they are depicted by the relative size of each word/descriptor. All four wines, irrespective of the frequency of punch downs, were perceived as drying, signifying they were perceived as equally astringent. This result is consistent with the comparable tannin levels on the wines of the second harvest, irrespective of the frequency of punch downs, up to Day 300 post-crushing (Figure 6E), which is when the sensory analysis took place. Further, a previous study reported that the tactile perception of dryness in Cabernet Sauvignon correlates with larger molecular weight tannins, a greater turbidity in their interaction with saliva, and overall higher tannin concentration [17], which is consistent with the present results. Whereas other subqualities of astringency changed little as a function of the treatments, especially in the 0 PD and 1 PD wines (Figure 9A and Figure 9B, respectively), the perception of bitterness increased concomitantly with the frequency of punch downs. Therefore, the 3 PD wines (Figure 9D) showed the highest bitterness perception. Wines produced with 2 PD (Figure 9C), on the other hand, were perceived as especially chalky. The higher bitterness perception in the 3 PD wines could not be directly explained by any of the phenolic parameters herein measured, which were indeed not greatly affected by the frequency of the punch-down treatments. However, monomeric flavan-3-ols, such as catechin and epicatechin, are known elicitors of bitterness in wines [18]. It is thus possible that the higher the frequency of punch downs in the 3 PD treatment may have led to higher flavan-3-ol extraction, thus explaining the higher perceived bitterness in these wines (though such a hypothesis may have to be analytically confirmed in future work). Finally, the present study analyzed these wines by CATA, a formal sensory technique that provides no hedonic liking/preference information. Future work may consider adding consumer preference data to studies of a similar scope.

## 3. Materials and Methods

### 3.1. Grapes and Vineyard Site

The present study spanned two consecutive but very different vintages in the Central Coast of California (USA), namely 2019 and 2020. Cabernet Sauvignon (clone 7), grafted on 1103P rootstock, was sourced from the Sunnybrook Ranch in the Paso Robles AVA of San Luis Obispo County (Paso Robles, CA, USA). Vines that sourced the fruit were drip-irrigated and trained into a split canopy system. The weather data for the calculation of cumulative growing degree days (GDD) was obtained from the UC IPM California Weather Data System (NCDC # 6730, Paso Robles, CA, USA). Fruit was manually harvested in 0.5-ton bins, for a total of 1.20 tons for each harvest, and these were immediately transported to the Research Winery of the Wine and Viticulture Department (California Polytechnic State University San Luis Obispo, CA, USA), with processing taking place the same day of fruit reception. In 2019, the grapes were harvested on 15 October, targeting 25 Brix. In 2020, two harvest points were established to target the contrasting maturity levels at 20 and 25 Brix, and harvest occurred on 18 September and 9 October, respectively. Forty clusters (n = 40) were randomly taken during each harvest prior to crushing, and they were hand-de-stemmed immediately to determine the Brix, pH, titratable acidity, yeast assimilable nitrogen, and potassium levels. Brix was measured with a handheld refractometer (Vee Gee Scientific, Kirkland, WA, USA). Titratable acidity was measured by titrating a known quantity of juice (5 mL) in a deionized water solution against 0.067 N of NaOH (Fisher Scientific, Waltham, MA, USA) to a pH endpoint of 8.2 [19]. Yeast assimilable nitrogen (YAN) and L-malic acid were measured enzymatically from the juice utilizing an analyzer (Y15 Automatic Analyzer, Admeo, Angwin, CA, USA) and commercially available kits (Biosystems, Barcelona, Spain).

### 3.2. Winemaking and Experimental Design

Cabernet Sauvignon grapes were processed using a crusher–destemmer (Bucher Vaslin, Niederweningen, Switzerland), keeping the rollers of the crusher disengaged. The musts were collected in individual 60 L plastic fermenters (Speidel, Swabia, Germany) (40 cm diameter and 60 cm height), with each fermenter receiving 50 kg (±0.1 kg) of must, which was recorded analytically on a scale (Adam Equipment, Oxford, NY, USA). Immediately after crushing, 50 mg/L of SO_2_ was added to each fermenter and incorporated with a gentle, 30 s punch down. Diammonium phosphate (Fermaid K, Lallemand, Rexdale, ON, Canada) was added to raise the yeast assimilable nitrogen to 300 mg/L prior to the alcoholic fermentation in all cases. The musts were inoculated with a commercial strain of *Saccharomyces cerevisiae* (EC-1118, Lallemand, Rexdale, ON, Canada) at a rate of 30 g/hL. Three contrasting cap management regimes were established: no punch down (0 PD), whereby the fermentors received no cap management during the fermentation/maceration period; and one, two, and three punch downs per day (namely, 1 PD, 2 PD, and 3 PD, respectively), whereby the fermenters received punch downs, each lasting one minute and separated by approximately 5 h in between, such as in the case of the 2 PD and 3 PD treatments. Punch downs were performed with a must plunger. All individual treatments were established in triplicate (n = 3), thus affording a total of 12 individual fermentations per harvest. The musts were inoculated with dry yeast (Enartis D20, Windsor, CA, USA) at a rate of 40 g/hL 6 h after crush. Total maceration time was set to 12 days. Malolactic bacteria *Oenococcus oeni* (VP41, Lallemand, QC, Canada) was added one day after yeast inoculation at a rate of 20 g/hL. The end of malolactic fermentation (MLF) was confirmed (≤0.2 g/L malic acid) by an enzymatic determination of L-malic acid (Y15 Automatic Analyzer, Admeo, Angwin, CA, USA) with commercially available kits (Biosystems, Barcelona, Spain), after which the wines received an addition of 25 mg/L of SO_2_. The wines were cold-stabilized at 5 °C for 45 days. The wines of the 2019 and 2020 vintages were bottled in March 2020 and April 2021, respectively, using DIAM 5 micro-agglomerated cork closure (G3 Enterprises, Modesto, CA, USA; oxygen transmission rate: 0.4 mg/bottle/year; oxygen initial release: 1.3 mg), which were stored in a vertical position and kept in controlled temperature (~10 to 12 °C) until analysis.

### 3.3. Wine Basic Chemical Composition

Wine titratable acidity (TA) and pH were measured following the same method detailed above for the determination of juice TA and pH. Ethanol (% *v*/*v*) was measured by near-infrared spectroscopy using a Alcolyzer Wine M/ME analysis system (Anton Paar, Graz, Austria). Acetic acid, glucose, fructose, malic acid, and lactic acid were determined enzymatically using commercial enzymatic analysis kits (Admeo, Biosystems Group, Hollister, CA, USA). The free and total SO_2_ concentrations were determined by the aspiration method [19].

### 3.4. Wine Spectrophotometric Analysis 

The spectrophotometric measurements included an analysis of the phenolic compounds and chromatic parameters. In the 2019 vintage, the wines were analyzed at pressing (Day 12) and after 1100 days post crush, which is equivalent to 3 years post-crushing. The wines of the 2020 vintage were analyzed throughout maceration (Days 6 and 12) and bottle aging, as well as up to 1110 days post-crushing (equivalent to 3 years post-crushing). Prior to analysis, the wine samples were centrifuged at 15,000× *g* in a microfuge (model 5415D; Eppendorf, Hamburg, Germany), and the supernatant was then transferred into clean 1 mL Eppendorf tubes prior to analysis. The anthocyanins and total polymeric pigments [herein defined as the sum of small polymeric pigments (SPP) and large polymeric pigments (LPP)], were measured as previously reported [20]. The tannins were analyzed by protein precipitation [21]. The CIELab color coordinates were determined in 1 mm path–length quartz cuvettes. The CIELab coordinates were calculated using Cary WinUV color software (version 6.0, Startek Technology, Boronia, VIC, Australia) under a D65 illuminant [22]. The spectrophotometric measurements were made with a Cary 60 UV–Vis spectrophotometer equipped with an 18-sample cell auto-sampler (Agilent Technologies, Santa Clara, CA, USA).

### 3.5. Wine Analysis by HPLC-Diode Array Detector-MS

The wines of both vintages were analyzed by a HPLC-diode array detector (DAD), with the peak identity confirmed by MS at selected stages of the winemaking process and up to 3 years of bottle aging. Prior to analysis, the wines were centrifuged for 10 min at 15,000× *g* (Eppendorf 5430 R, Hamburg, Germany) and filtered through a 0.45 μm membrane (Sartorius, Goettingen, Germany). The wines were analyzed in an Agilent 1100 series HPLC system coupled to a DAD (Agilent Technologies), as previously described [23], with minor modifications. Separation occurred in a Zorbax SB-C18 column (4.6 mm × 150 mm, 3.5 μm particle size; Agilent Technologies), which was thermostated at 40 °C and protected by a guard column of the same packing material. Peak identity was confirmed using a Waters Acquity I-Class ultra-performance liquid chromatography system that was connected to an AB Sciex 4000 Q-Trap MS/MS (Waters, Milford, MA, USA). The column eluent, under the same conditions described earlier, was directed to the mass spectrometer that was operated in positive ionization mode, and the compounds were detected by multiple reaction monitoring. Monomeric anthocyanins were quantified using malvidin-3-glucoside chloride as standard (Extrasynthèse, Lyon, France) and a standard calibration curve (R^2^ = 0.99). To facilitate the interpretation of the results, the pigments were grouped as glycosides (i.e., monoglucosylated anthocyanins, including delphinidin-, cyanidin-, petunidin-, peonidin-, and malvidin-3-glucosides); acylated (i.e., acylated and coumaroylated anthocyanin monoglucosides); vitisins (i.e., pyranoanthocyanins, including Vitisin A and Vitisin); and polymeric pigments.

### 3.6. Sensory Analysis

Panelist availability and limited resources precluded the analysis of all the three sets of wines. Therefore, the wines of the second harvest of the 2020 vintage were selected for sensory analysis because they were deemed the closest to a typical commercial harvest for Premium Cabernet Sauvignon in the Paso Robles AVA of the Central Coast of California. The wines were analyzed after 5 months of bottle aging using the sensory technique Check-All-That-Apply (CATA), which allowed for the binary data to be extracted, and they were primarily based on attribute presence/absence. Thirteen panelists (three males and ten females) between 21 to 60 years of age participated in this study. Other than varietals, no information about the nature of the study was provided to reduce bias. The Cal Poly Institutional Review Board for human subject participation approved the project (protocol number: 2021-058). All individuals were screened for visual disorders using Ishihara maps and for bitterness sensitivity using 6-n-propyl-thiouracil (PROP). The results of these tests indicated that none of the panelists had color deficiencies and that the panel was composed of 15% non-tasters, 62% medium tasters, and 23% super tasters. The panelists were provided and tracked with a random three-digit code that would serve as their identifier in the formal analysis section. The panelists participated in four 60 min training sessions over the course of two consecutive weeks, during which the panelists discussed, agreed upon, and trained with reference standards (Supplementary Materials Table S1), as well as practiced evaluating the wines. The panelists were instructed to only evaluate the taste and astringency, and, after deliberation the attributes selected included bitterness, dryness/drying, juicy, chalky/powdery, velvety, sandy, and suede—a total of 7 attributes (Supplementary Materials Table S1). All the standards were produced in-house and contained specified ingredients, base, procedure, and instructions (Supplementary Materials Table S1). Both high and low intensity standards for bitterness and astringency were prepared. Four of the standards were presented as touch standards. All the standards were reviewed by the panelists during the training sessions to calibrate them and to ensure they accurately reflected the sensory attribute they were intended to simulate. The panelists formally evaluated the experimental wines in three 60 min sessions in individual sensory booths lit with incandescent lightening (General Electric: Crystal clear 40W, Boston, MA, USA). RedJade Sensory Software (RedJade Sensory Software, Tragon Corporation, Palo Alto, CA, USA (https://redjade.net/sensory-evaluation-software/)), determined the random order, using a Latin Square Design, in which the wines were monadically presented to each panelist. Redjade also generated four-digit random codes that were labeled on the bottom of each regulation ISO glass. The panelists received 30 mL aliquots of wine served at room temperature with five unsalted crackers (Nabisco, East Hanover, NJ, USA), a water bottle (Lake Arrowhead Spring Water, Blue Triton, Samford, CT, USA), and a spit cup. Panelists were instructed to wait 1 min, eat a cracker, and drink water between samples. This was conducted to allow for a palate cleanse before the next wine. Once panelists finished their sixth wine, they were given a five-minute break. They were instructed to rest by eating crackers and drinking water. All panelists attended every session. All the sensory data were collected by RedJade Sensory Software. 

### 3.7. Statistical Analysis

Wines were produced in triplicate fermentations (n = 3) across all the three vintages under study. The fruit data were analyzed by a fixed-effect one-way analysis of variance (ANOVA). The basic chemical, phenolic, HPLC data, and chromatic composition of the wines was analyzed by a one-way ANOVA. In all cases, Fisher’s LSD test was used as a post hoc comparison of the means with a 5% level for rejection of the null hypothesis. The data were analyzed with XLSTAT (Addinsoft, Version 2023.1414, Paris, France), and all the graphical representations were prepared with GraphPad Prism software version 9.0 (GraphPad Software, San Diego, CA, USA). The sensory data were collected by RedJade Sensory Software (RedJade Sensory Software, Tragon Corporation, Palo Alto, CA, USA) and analyzed using the Word Cloud function of XLSTAT v. 2022 (Addinsoft, Paris, France). 

## 4. Conclusions

**Figure 10:**
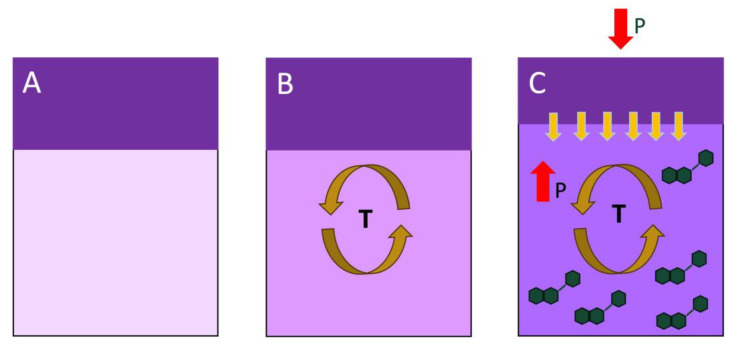
The proposed mechanism for the dynamics of phenolic extraction in conditions of minimal cap management/physical mixing. (**A**) The initial stage of cap formation at relatively low temperatures; (**B**) convective mixing aided by temperature (T); and (**C**) cap compaction due to upward (red arrow, P) (CO_2_) and downward (atmospheric) pressure (red arrow, P), coupled with convective mixing aided by temperature.

The present study explored the effect of punch-down frequency on the chromatic, phenolic, and sensory composition of Cabernet Sauvignon wines throughout winemaking and after an extensive period of bottle aging. The experiments were repeated over two contrastingly different consecutive harvests: one (2019) colder than average and the other (2020) warmer than average. Results herein discussed were obtained in and thus confined to fermentors of a moderate volume, but they can shed light into the dynamics of extraction of sensory-active compounds during winemaking. There were almost non-existent effects when varying the frequency of punch downs on parameters such as ethanol, pH, titratable acidity, lactic acid, and glucose + fructose. In 2019, the chromatic and phenolic differences between different PD regimes were relatively minor. Initially, during fermentation, higher levels of all anthocyanin classes were observed in the 1 PD wines and the lowest levels in the 0 PD wines. It is possible that during the early stages of the maceration process, in conditions of relatively low temperature (and thus low convective mixing), phenolic extraction may occur at a relatively low rate in an undisturbed cap. Under such conditions, disrupting the cap by physical mixing will aid phenolic extraction (Figure 10A). This may explain why there was an initially lower phenolic extraction in the 0 PD wines relative to treatments that received punch downs; however, these differences subsided later once the convective effect of temperature started regulating the phenolic extraction. In addition, the general anthocyanin content of the wines of the first harvest (unripe) was on average 27% higher than that of the wines of the second harvest (ripe), perhaps reflecting the warmer 2020 vintage. Again, these differences disappeared after 3 years of bottle aging irrespective of the vintage and harvest date. Acylated anthocyanins were preferentially lost during the aging, particularly in the 2019 wines. In 2020, the general polymeric pigment content of the wines of the second harvest was higher than that in the wines of the first harvest, with the 3 PD wines showing higher polymeric pigments and yellow hues than the 0 and 2 PD wines after 3 years of bottle aging. These results point to an overall minor effect of the frequency of punch downs on the phenolic and chromatic composition of the wines, which sits at odds with empirical winemaking observations. That is, that a higher frequency of physical mixing during maceration should lead to higher phenolic extraction. Herein, we propose an explanation for the lower-than-expected phenolic extraction in frequent mixing conditions, as well as for the higher-than-expected phenolic extraction in low frequency of mixing conditions. We suggest that a higher frequency of mixing past a certain threshold may lead to phenolic losses due to chemical and or enzymatic oxidations, as well as phenolic losses due to adsorption and absorption into fermentation solids. Continuously disrupting the cap in small volume fermentors may also release the pressure built within the fermentation solids atop of the fermentor, thus potentially downregulating the phenolic extraction. However, phenolic extraction in the absence of cap management still needs to be explained. Figure 10 presents a possible mechanistic explanation for phenolic extraction under limited cap management conditions, i.e., under low or absence of physical mixing conditions during maceration. In fermentors of relatively small volume, under sufficiently high fermentation temperature, convective movements may facilitate physical mixing, thereby allowing for sufficient phenolic extraction in an undisturbed cap (Figure 10B). Furthermore, as fermentation progresses, an undisturbed cap may build more pressure than a disrupted one, thus upregulating the phenolic extraction within the cap under pressure, with the aided convective mixing of the temperature (Figure 10C). However, it should be noted that minimal cap management may imply lower rates of oxygen dissolution during alcoholic fermentation, which can bear negative consequences, especially in relation to the production of SH_2_ and other volatile sulfur compounds associated with reduction aromas. We propose that the measurement of the evolution of the oxidation–reduction potential (ORP) of fermentations under such contrasting cap management conditions should be of special interest for winemakers. For example, under conditions of minimal cap disruption and sufficient temperature, phenolic extraction may be favored, as herein shown, and the monitoring of the ORP may inform about the timing of oxygen additions to avoid the production of SH_2_ and other volatiles associated with reduction flavors and aromas. Another observation is that, in the present study, the degree of the initial crushing of the grapes was relatively low (as the rollers were disengaged). It would be thus interesting to test a similar experimental set up with berries that have undergone a more through degree of crushing.

Sensory analysis of the second harvest of the 2020 wines showed that the wines of all four PD regimes were perceived as drying, signifying they were perceived as equally astringent, which was consistent with the tannin levels in said wines. The perception of bitterness increased concomitantly with the frequency of punch downs; thus, the 3 PD wines showed the highest bitterness perception.

Overall, present results suggest that, in sufficiently warm fermentations and small volumes, phenolic extraction occurs regardless of fruit maturity (at least within the ripeness levels assessed in the present study), even under conditions of minimal cap management.

## Figures and Tables

**Table 1:** The basic chemical composition of the Cabernet Sauvignon grapes from the 2019 and 2020 vintages at harvest. Average values (n = 3) followed by the standard error of the mean.

Vintage	Harvest Date	Brix	pH	Titratable Acidity (g/L Tartaric Acid)	Malic Acid (g/L)	Yeast Available Nitrogen (mg/L)	Potassium (mg/L)
2019	10/15/2019	25.3 ± 0.05	3.63 ± 0.01	6.70 ± 0.05	1.06 ± 0.12	182 ± 13	2020 ± 100
2020	9/18/2020	20.9 ± 0.07	3.55 ± 0.02	5.32 ± 0.11	0.91 ± 0.14	194 ± 25	2230 ± 90
	10/9/2020	24.7 ± 0.09	3.76 ± 0.02	4.20 ± 0.02	0.46 ± 0.03	177 ± 18	2405 ± 35

**Table 2:** One-way ANOVA of the basic chemical composition of Cabernet Sauvignon wines made with contrasting punch-down regimes and two ripeness levels in 2020. Average values followed by the standard error of the mean (n = 3).

Harvest	Winemaking Treatment	Ripeness Level	Alcohol % (*v*/*v*)	pH	Titratable Acidity (g/L)	Malic Acid (g/L)	Lactic Acid (g/L)	Acetic Acid (g/L)	Glucose + Fructose (g/L)
2019	0 PD	Ripe	14.22 ± 0.01 a	3.94 ± 0.00 a	5.12 ± 0.04 a	0.05 ± 0.00 a	1.39 ± 0.02 a	0.34 ± 0.02 b	0.33 ± 0.02 a
	1 PD	Ripe	14.11 ± 0.01 a	3.99 ± 0.01 a	5.37 ± 0.11 a	0.03 ± 0.03 b	1.37 ± 0.00 a	0.43 ± 0.04 a	0.31 ± 0.05 a
	2 PD	Ripe	13.91 ± 0.01 a	3.93 ± 0.00 a	5.26 ± 0.03 a	0.04 ± 0.00 ab	1.40 ± 0.02 a	0.42 ± 0.03 a	0.27 ± 0.03 a
	3 PD	Ripe	13.86 ± 0.01 a	3.95 ± 0.00 a	5.14 ± 0.04 a	0.00 ± 0.00 c	1.38 ± 0.01 a	0.44 ± 0.02 a	0.31 ± 0.00 a
	*p*-value		0.135	0.063	0.127	**0.001 b**	0.830	**0.004**	0.373
2020	0 PD	Unripe	11.73 ± 0.04 a	3.95 ± 0.02 a	4.82 ± 0.16 a	0.05 ± 0.00 a	1.21 ± 0.21 a	0.45 ± 0.01 a	0.23 ± 0.06 a
	1 PD	Unripe	11.89 ± 0.02 a	3.94 ± 0.01 a	4.74 ± 0.35 a	0.04 ± 0.01 a	1.21 ± 0.17 a	0.38 ± 0.02 a	0.03 ± 0.06 a
	2 PD	Unripe	11.82 ± 0.02 a	3.94 ± 0.01 a	4.82 ± 0.17 a	0.03 ± 0.04 a	1.16 ± 0.07 a	0.41 ± 0.02 a	0.03 ± 0.02 a
	3 PD	Unripe	11.74 ± 0.04 a	3.94 ± 0.04 a	4.84 ± 0.22 a	0.02 ± 0.00 b	1.19 ± 0.11 a	0.41 ± 0.04 a	0.03 ± 0.01 a
	*p*-value		0.168	0.752	0.239	**0.012**	0.424	0.279	0.219
	0 PD	Ripe	12.55 ± 0.03 a	3.89 ± 0.06 a	4.49 ± 0.22 a	0.04 ± 0.02 a	0.63 ± 0.11 a	0.37 ± 0.04 a	0.11 ± 0.03 a
	1 PD	Ripe	12.60 ± 0.02 a	3.73 ± 0.04 a	5.77 ± 0.12 a	0.01 ± 0.02 a	1.23 ± 0.12 a	0.52 ± 0.04 a	0.07 ± 0.01 a
	2 PD	Ripe	12.56 ± 0.03 a	3.77 ± 0.06 a	5.12 ± 0.32 a	0.01 ± 0.02 a	1.04 ± 0.02 a	0.46 ± 0.02 a	0.08 ± 0.01 a
	3 PD	Ripe	12.40 ± 0.02 a	3.77 ± 0.04 a	5.13 ± 0.22 a	0.00 ± 0.00 a	0.91 ± 0.13 a	0.38 ± 0.04 a	0.07 ± 0.01 a
	*p*-value		0.330	0.562	0.523	0.280	0.638	0.138	0.919

(a) Different letters within a given column for a given vintage or ripeness level indicate the significant differences established by Tukey’s HSD test, with a significance level of *p* < 0.05. (b) Significant *p*-values (<0.05) are shown in bold fonts.

## Data Availability

The data presented in this study are available in this article and in the Supplementary Materials.

## Supplementary Materials

The following supporting information can be downloaded at: https://www.mdpi.com/article/10.3390/molecules29112509/s1, Table S1: Ingredients and specifications used in the CATA analysis of Cabernet Sauvignon wines during the panel trainings and formal evaluations. Figure S1: Evolution of growing degree days for the Paso Robles AVA of California (USA), and harvest dates for the 2019 and 2020 vintages and the 2011–2022 average.
