# Supplementary material for: Effect of Cap Management Frequency on the Phenolic, Chromatic, and Sensory Composition of Cabernet Sauvignon Wines from the Central Coast of California over Two Vintages
Source: Molecules. 2024 May 26;29(11):2509. doi: 10.3390/molecules29112509 (PMC11173441; doi:10.3390/molecules29112509)
Supplement: Supplementary file 1 [file molecules-29-02509-s001.zip › molecules-3003397-supplementary.pdf]

Supplemental Table S1. Ingredients and specifications used in the CATA analysis of Cabernet Sauvignon wines during the panel trainings and formal evaluations.

Standard	Category	Ingredients	Base	Procedure and instructions
Bitterness - High intensity	Taste	4 g Caffeine (NuSci)	1200 mL Tap water	Mix until homogenized.
Bitterness - Low intensity	Taste	2 g Caffeine (NuSci)	1200 mL Tap water	Mix until homogenized.
Astringency - High intensity	Mouthfeel	4 g Tannic Acid (Sigma Aldrich)	1200 mL Tap water	Mix until homogenized.
Astringency - Low intensity	Mouthfeel	1 g Tannic Acid (Sigma Aldrich)	1200 mL Tap water	Mix until homogenized.
Dryness/drying	Mouthfeel	2 g Alum (McCormick)	1200 mL Tap water	Mix until homogenized.
Juicy	Mouthfeel	Apple Juice (Motts 100% Apple Juice), 6 g Tartaric Acid (Fisher Chemical)	1200 mL Apple Juice (Mott's)	Mix until homogenized.
Chalky/powdery	Mouthfeel	Smarties (Smarties Candy Company)		Finely crushed Smarties and placed into Ziplock bags.
Velvety	Mouthfeel	Dress (Willow Ridge)	Canvas Board	Cut two 5 x 12.7 cm pieces. Glued one to canvas. Pulled one through canvas hole and allowed to dangle.
Sandy	Mouthfeel	Sand (Mitchell Park)		Collected sand from park and placed into Petri dishes.
Suede	Mouthfeel	Jacket (Chico's)	Canvas Board	Cut two 5 x 12.7 cm pieces. Glued one to canvas. Pulled one through canvas hole and allowed to dangle.

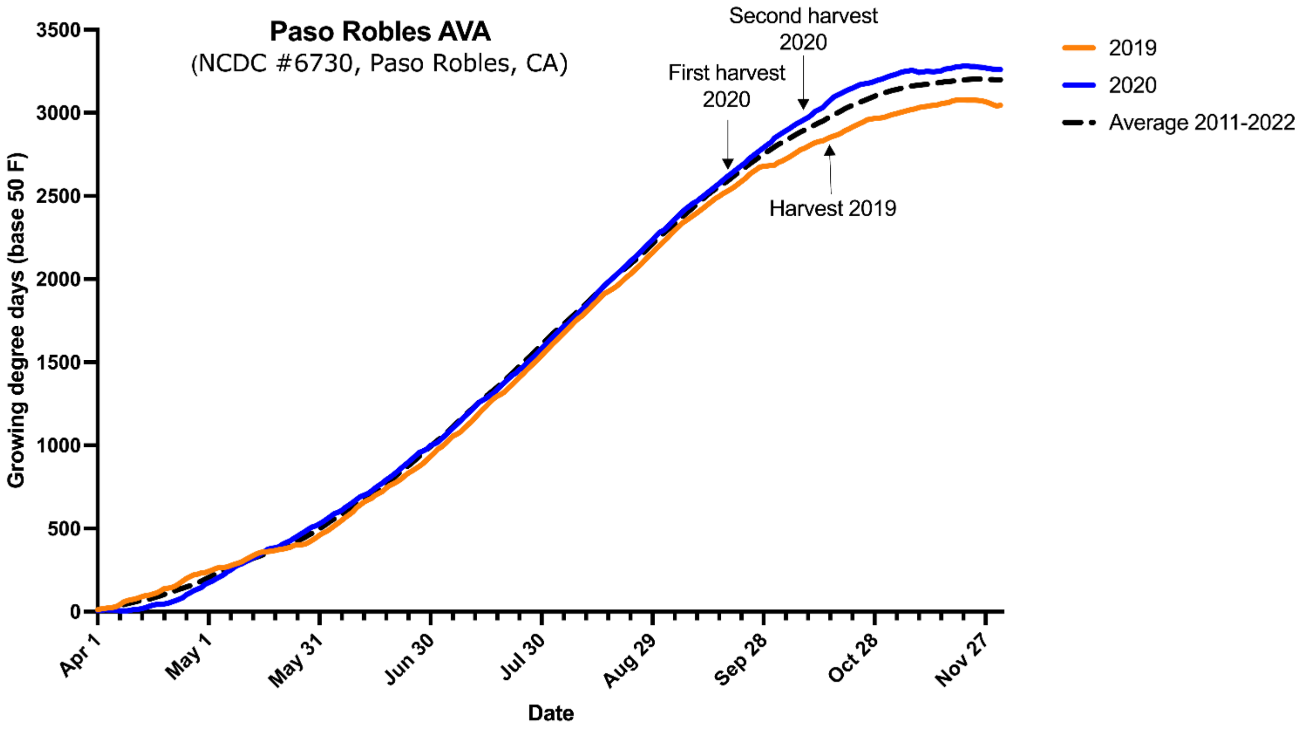

Supplemental Figure S1. Evolution of growing degree days for the Paso Robles AVA of California (USA), and harvest dates for the 2019 and 2020 vintages and the 2011-2022 average.
